# Association Between Gut Microbiota Diversity and Body Mass Index (BMI) in Healthy Young Adults in the United States: Insights Into the Gut-Brain-Metabolic Axis Using the Curated Metagenomic Data

**DOI:** 10.7759/cureus.97746

**Published:** 2025-11-25

**Authors:** Nenrot S Gopep, Abosede O Odukale-Okuneye, Ifelunwa M Osanakpo, Hillary C Ugwu, Omotayo O Amure, Kashif A Khan, Ozioma Grace Obaisi, Ngozi T Akueme, Akintunde C Akinboboye, Ifesinachi Nwankwor, Chibuzo N Nwodo

**Affiliations:** 1 Community Medicine, Federal Medical Center, Keffi, NGA; 2 Public Health, Georgia Southern University, Statesboro, USA; 3 Stroke Medicine, Sherwood Forest Hospitals NHS Foundation Trust, Sutton-in-Ashfield, GBR; 4 Internal Medicine, Carle Foundation Hospital, Urbana, USA; 5 Intensive Care Unit, Prince Mutaib bin Abdulaziz Hospital, Sakaka, SAU; 6 Family Medicine, Lagos State University Teaching Hospital, Lagos, NGA; 7 Family Medicine, Markham Road Medical Centre, Markham, CAN; 8 Internal Medicine, Diana, Princess of Wales Hospital, Grimsby, GBR; 9 Internal Medicine, One Brooklyn Health, Brooklyn, USA; 10 Emergency Medicine, University of Medical Sciences Teaching Hospital, Ondo, NGA; 11 General Medicine, General Hospital Agulu, Agulu, NGA; 12 Ear, Nose, and Throat (ENT), Royal Derby Hospital, Derby, GBR

**Keywords:** alpha-diversity, body mass index, gut–brain–metabolic axis, gut microbiota, metagenomics, young adults

## Abstract

Background: Emerging evidence suggests that gut microbiota diversity plays a critical role in metabolic regulation and may influence body mass index (BMI). However, findings in healthy populations remain inconsistent.

Objective: This study aims to determine whether gut microbiota alpha-diversity is associated with BMI among healthy young adults aged 18-39 years in the United States and to explore potential implications for the gut-brain-metabolic axis.

Methods: This cross-sectional study utilized publicly available metagenomic data from the CuratedMetagenomicData repository. After preprocessing in R version 4.5.0 (R Foundation for Statistical Computing, Vienna, Austria), data were analyzed using Stata version 18 (Released 2023; StataCorp LLC, College Station, TX). Alpha-diversity indices (Shannon, Simpson, and Richness) were computed and examined across BMI categories (normal, overweight, and obese) using one-way analysis of variance (ANOVA) and chi-square tests. Linear regression models were employed to assess associations between BMI and diversity measures, adjusting for age and gender.

Results: Among 147 participants, BMI differed significantly across weight categories (p < 0.001), but no significant association was observed between Shannon diversity and BMI (p = 0.527). Age emerged as the only significant predictor of BMI in adjusted models (p < 0.001).

Conclusion: Gut microbial alpha-diversity was not significantly associated with BMI among healthy young adults. Functional microbial characteristics, rather than diversity alone, may better explain variations in metabolic status.

## Introduction

The microbiota of the human gut, a complex of microorganisms that inhabit the gastrointestinal tract, has become a central modulator of metabolic health, with effects on physiological processes, energy, immune response, and neurobehavioral response via the gut-brain axis [1]. Recent developments in metagenomics have enabled the deep study of gut microbiota structure and composition, which has shed light on how it correlates with body mass index (BMI), obesity, and metabolic disease [2-3]. Alpha-diversity, a microbial evenness and richness measure of a single community, has been associated with efficiency in energy harvesting, inflammation, and insulin sensitivity [4]. A drop in microbial diversity can be frequently found in people with increased BMI, which signifies a break in metabolic and immune homeostasis that can be one of the factors of developing obesity-related pathophysiology [5].

The gut-brain-metabolic axis is a new conceptual framework of the two-way communication between the gut microbiota, central nervous system (CNS), and metabolic organs [6-7]. Neural and endocrine mechanisms intermediate neuroactive products of gut microbes, including short-chain fatty acids (SCFAs), serotonin precursors, and gamma-aminobutyric acid (GABA), have an impact on appetite control, stress response, and energy expenditure [8]. A change in microbial diversity can thus interfere with neuroendocrine signaling, which is one of the causes of metabolic dysregulation and obesity [9-10]. For example, dysbiosis can interfere with hypothalamic signaling of satiety, leading to the intake of more food and metabolic lipid problems. Additionally, the neuroimmune aspect of metabolic health is emphasized by the use of microbiota-derived metabolites to regulate systemic inflammation, a major causative connection between obesity and insulin resistance, and cardiovascular disease [11-12].

The relationship between metabolic phenotypes and gut microbial diversity has been shown to be highly correlated by both human and animal studies [13]. Lean persons are more likely to have a balanced system of Bacteroidetes and Firmicutes dominant microbial ecosystem, in contrast to obese persons with lower diversity and the propensity to obtain energy-yielding microbial species [14-15]. These microbial ecosystems are further influenced by environmental and lifestyle factors, including diet, physical activity, antibiotic exposure, and stress, making it clear that both external factors and internal control via the gut-brain axis interact [16]. Nevertheless, in spite of these outcomes, the existing body of evidence on the relationships between gut microbiota diversity and BMI among young and healthy adults is still scarce, which is a demographic group that has been largely neglected in studies of metabolic disorders [17-18].

The CuratedMetagenomicData repository provides a rare chance to study this relationship based on harmonized profiles of human gut microbiomes associated with phenotypic/demographic information [19-20]. Using this dataset, the researchers are able to evaluate how changes in microbial alpha-diversity are associated with BMI in nonclinical populations, and this will illuminate the changes in metabolism that occur before clinical disease [21]. These insights may help promote the knowledge of how microbiome diversity is a biomarker and a possible modifiable determinant of metabolic resilience.

The main objective of the study is to determine whether gut microbiota alpha-diversity is associated with BMI among healthy young adults aged 18-39 years in the United States, using metagenomic data from the CuratedMetagenomicData repository, and to explore potential implications for the gut-brain-metabolic axis. The results may be used to guide the prevention measures related to the development of microbiome regulation aimed at preserving the equilibrium in the metabolic processes and minimizing the chances of obesity and associated conditions over time.

## Materials and methods

Study design and data source

This study employed a cross-sectional design to investigate the association between gut microbiota diversity and BMI using publicly available metagenomic data from the CuratedMetagenomicData repository [20]. The repository compiles curated and standardized human microbiome datasets generated from shotgun metagenomic sequencing, with unified processing pipelines that ensure cross-study comparability. Data were obtained from the Human Microbiome Project (HMP, 2012), a large-scale initiative that collected microbial and phenotypic data from healthy individuals.

The dataset was retrieved and preprocessed in the R environment version 4.5.0 (R Foundation for Statistical Computing, Vienna, Austria), where extraction, filtering, and merging procedures were conducted to ensure data completeness, uniformity, and analytical consistency. Stool samples were selected, variable names were harmonized, and microbiome abundance tables were merged with corresponding sample metadata.

Study population

The study population comprised healthy young adult participants enrolled in the Human Microbiome Project whose stool metagenomic data and corresponding metadata were available within the CuratedMetagenomicData repository. After rigorous preprocessing steps, including the exclusion of incomplete cases, filtering of non-stool body sites, and merging of relevant datasets, the final analytic sample included adults aged 18-39 years from the United States. Individuals with missing or implausible BMI values, incomplete demographic information, or non-stool microbiota profiles were excluded. This restricted cohort ensured internal validity and allowed a focused assessment of the association between gut microbial diversity and BMI within a metabolically stable age range.

Variables and measures

The primary outcome variable was BMI, computed as weight in kilograms divided by height in meters squared (kg/m²). BMI was treated as a continuous variable and further categorized using World Health Organization (WHO) cutoffs: underweight (<18.5 kg/m²), normal weight (18.5-24.9 kg/m²), overweight (25.0-29.9 kg/m²), and obese (≥30 kg/m²). The key exposure variable was gut microbiota alpha-diversity, quantified using the Shannon diversity index, derived from species-level taxonomic abundance profiles. Additional diversity measures, including the Simpson and Richness indices, were also evaluated for descriptive purposes. Covariates included age (in years) and gender, which were incorporated as potential confounders in the adjusted regression models.

Statistical analysis

Data were analysed using Stata version 18 (Released 2023; StataCorp LLC, College Station, TX, USA). Continuous variables, including BMI and microbiota diversity indices (Shannon, Simpson, and Richness), were summarized using means and standard deviations, while the categorical variable gender was presented as frequencies and percentages. Descriptive statistics were stratified by BMI categories (underweight, normal weight, overweight, and obese) to explore distributional patterns across groups. Group differences in continuous variables across BMI categories were assessed using one-way analysis of variance (ANOVA). Specifically, mean comparisons were computed for BMI, Shannon diversity, Simpson index, and age across BMI categories. The variability of estimates was assessed using standard deviations, and results were presented. Associations between categorical variable (gender) and BMI categories were examined using the chi-square (χ²) test of independence, with row percentages reported to facilitate interpretation.

Pearson correlation analysis was performed to assess the linear relationship between BMI and microbiota diversity indices. Simple and multiple linear regression models were fitted to estimate the strength and direction of association between Shannon diversity and BMI. The multiple regression model was adjusted for potential confounders, including age and gender, to control for demographic variation.

Multicollinearity was assessed using the variance inflation factor (VIF), which ranged between 1.03 and 1.04, with a mean VIF of 1.03, indicating no significant multicollinearity among the predictors. Statistical significance was determined at a two-sided p < 0.05.

Missing data

During preprocessing, missing and incomplete records were identified primarily within demographic and sequencing metadata fields. Observations lacking essential variables such as BMI, age, gender, or microbiota diversity indices were excluded from the analytical dataset to maintain data integrity. The rigorous filtering and merging procedures reduced the sample size but ensured that all retained records had complete information for the primary exposure and outcome measures. No imputation methods were applied, and all analyses were performed on complete cases only.

Ethical considerations

The study utilized publicly available, de-identified metagenomic and metadata from the CuratedMetagenomicData repository. Since no individual-level identifiers were accessible and the data were originally collected under institutional ethical approvals as part of the Human Microbiome Project, additional ethical clearance was not required for this secondary analysis. The research complied with the principles outlined in the Declaration of Helsinki and adhered to responsible data use and confidentiality standards in handling open-access human microbiome datasets.

## Results

A total of 147 healthy adults aged 18-39 years from the United States were included in the final analytic sample. Table 1 summarizes the demographic and microbiota diversity characteristics of the study population across BMI categories (normal weight, overweight, and obese).

**Table 1:** Participant characteristics and gut microbiota diversity across BMI categories ANOVA: analysis of variance; BMI: body mass index; SD: standard deviation Values are presented as mean ± SD for continuous variables and as n (%) for categorical variables. F-statistics derived from one-way ANOVA for continuous variables; χ²-statistics derived from Pearson’s chi-square test for categorical variables

Variable	Normal (n = 78)	Overweight (n = 52)	Obese (n = 17)	Test statistic	p-value
Age (years) (mean ± SD)	25.48 ± 4.31	26.42 ± 4.92	31.35 ± 5.47	F = 11.01	<0.001
BMI (kg/m²) (mean ± SD)	22.14 ± 1.59	26.38 ± 1.29	31.11 ± 1.11	F = 326.55	<0.001
Shannon diversity (mean ± SD)	2.31 ± 0.54	2.43 ± 0.48	2.32 ± 0.64	F = 0.90	0.411
Simpson diversity (mean ± SD)	0.80 ± 0.14	0.84 ± 0.10	0.82 ± 0.12	F = 1.30	0.275
Gender(n, %)	-	-	-	χ² = 0.69	0.708
Male	46 (56.10%)	27 (32.93%)	9 (10.98%)	-	-
Female	32 (49.23%)	25 (38.46%)	8 (12.31%)	-	-

From the findings above (Table 1), it’s evident that the mean age differed significantly among the three BMI groups (F = 11.01, p < 0.001), with obese participants being notably older (31.35 ± 5.47 years) than their normal-weight (25.48 ± 4.31 years) and overweight (26.42 ± 4.92 years) counterparts. As expected, BMI increased progressively and significantly across categories (F = 326.55, p < 0.001), confirming the accuracy of group classification.

In contrast, measures of gut microbial alpha-diversity, including the Shannon and Simpson diversity indices, did not differ significantly across BMI groups. The mean Shannon index was 2.31 ± 0.54 in the normal-weight group, 2.43 ± 0.48 among overweight individuals, and 2.32 ± 0.64 in the obese group (F = 0.90, p = 0.411). Similarly, Simpson diversity scores were comparable across groups (F = 1.30, p = 0.275), suggesting that overall microbial richness and evenness were relatively stable regardless of BMI.

The gender distribution was balanced across categories, with males representing 46 (56.1%) of normal weight, 27 (32.9%) of overweight, and nine (11.0%) of obese participants. Females comprised 32 (49.2%), 25 (38.5%), and eight (12.3%) of these respective groups. The association between gender and BMI classification was not statistically significant (χ² = 0.69, p = 0.708), indicating no significant relationship with BMI. Taken together, these results suggest that while BMI and age varied significantly among study participants, microbial alpha-diversity measures remained largely consistent across BMI categories. Table 2 presents pairwise Pearson’s correlation that explores the relationship between BMI and measures of gut microbial alpha-diversity (Shannon, Simpson, and Richness indices).

**Table 2:** Pearson’s correlation coefficients between body mass index and gut microbiota diversity indices Values represent Pearson’s correlation coefficients (r) between study variables, with corresponding p-values shown in parentheses. (-) Intentionally left blank

Variables	BMI	Shannon	Simpson	Richness
BMI	1.000	-	-	-
Shannon	0.060	1.000	-	-
-	(0.471)	-	-	-
Simpson	0.090	0.913	1.000	-
-	(0.279)	(0.000)	-	-
Richness	-0.088	0.704	0.471	1.000
-	(0.290)	(0.000)	(0.000)	

The correlation analysis in Table 2 above revealed no significant linear association between BMI and any of the gut microbial diversity measures. Specifically, BMI was weakly and non-significantly correlated with the Shannon index (r = 0.06, p = 0.471), Simpson index (r = 0.09, p = 0.279), and microbial richness (r = -0.09, p = 0.290). In contrast, the alpha-diversity metrics were strongly interrelated, as expected. Shannon and Simpson indices exhibited a very high positive correlation (r = 0.91, p < 0.001), while Shannon diversity also correlated positively with microbial richness (r = 0.70, p < 0.001). Similarly, Simpson diversity demonstrated a moderate positive correlation with richness (r = 0.47, p < 0.001).

These findings suggest that although alpha-diversity metrics are internally consistent indicators of microbial composition, they do not show a measurable linear relationship with BMI within this relatively healthy young adult cohort. The absence of significant correlations indicates that BMI variation in this population is unlikely to be driven by overall microbial diversity alone, emphasizing the need for further analyses exploring more refined microbial or functional shifts associated with adiposity.

To further examine whether gut microbial alpha-diversity predicts variation in BMI, linear regression models were constructed with BMI as the dependent variable and the Shannon diversity index as the key predictor. The unadjusted and age and sex adjusted models are summarized in Table 3.

**Table 3:** Linear regression analysis of the association between gut microbiota diversity and body mass index BMI: body mass index; *** p < 0.00; (-) intentionally left blank The table displays unstandardized beta coefficients (β) and standard errors in parentheses from linear regression models examining the association between Shannon diversity and BMI. Model 1 presents the unadjusted association, while model 2 adjusts for age and gender

Variable	Model 1	Model 2
Shannon	0.381	0.018
-	(0.527)	(0.500)
Age(in years)	-	0.256^***^
-	-	(0.053)
Female	-	-0.202
-	-	(0.530)
Constant	23.782^***^	17.945^***^
-	(1.273)	(1.785)
Observations	147	147
R^2^	0.004	0.147

In model 1, Shannon diversity showed a small, non-significant positive association with BMI (β = 0.381, p = 0.527). After adjusting for age and gender as shown in model 2, the association remained non-significant (β = 0.018, p = 0.500), indicating that microbial diversity was not an independent predictor of BMI within this cohort. Conversely, age emerged as a significant covariate (β = 0.256, p < 0.001), suggesting that BMI tended to increase modestly with age among participants. Gender was not associated with BMI after adjustment (β = −0.202, p = 0.530).

The adjusted model explained approximately 14.7% of the variance in BMI (R² = 0.147), compared to less than 1% in the unadjusted model, emphasizing the contribution of demographic covariates rather than microbial diversity to BMI variability in this population of healthy adults aged 18-39 years. Overall, these findings suggest that, while gut microbial alpha-diversity is an integral ecological measure, it may not independently account for BMI differences among relatively young and metabolically healthy individuals.

**Figure 1:**
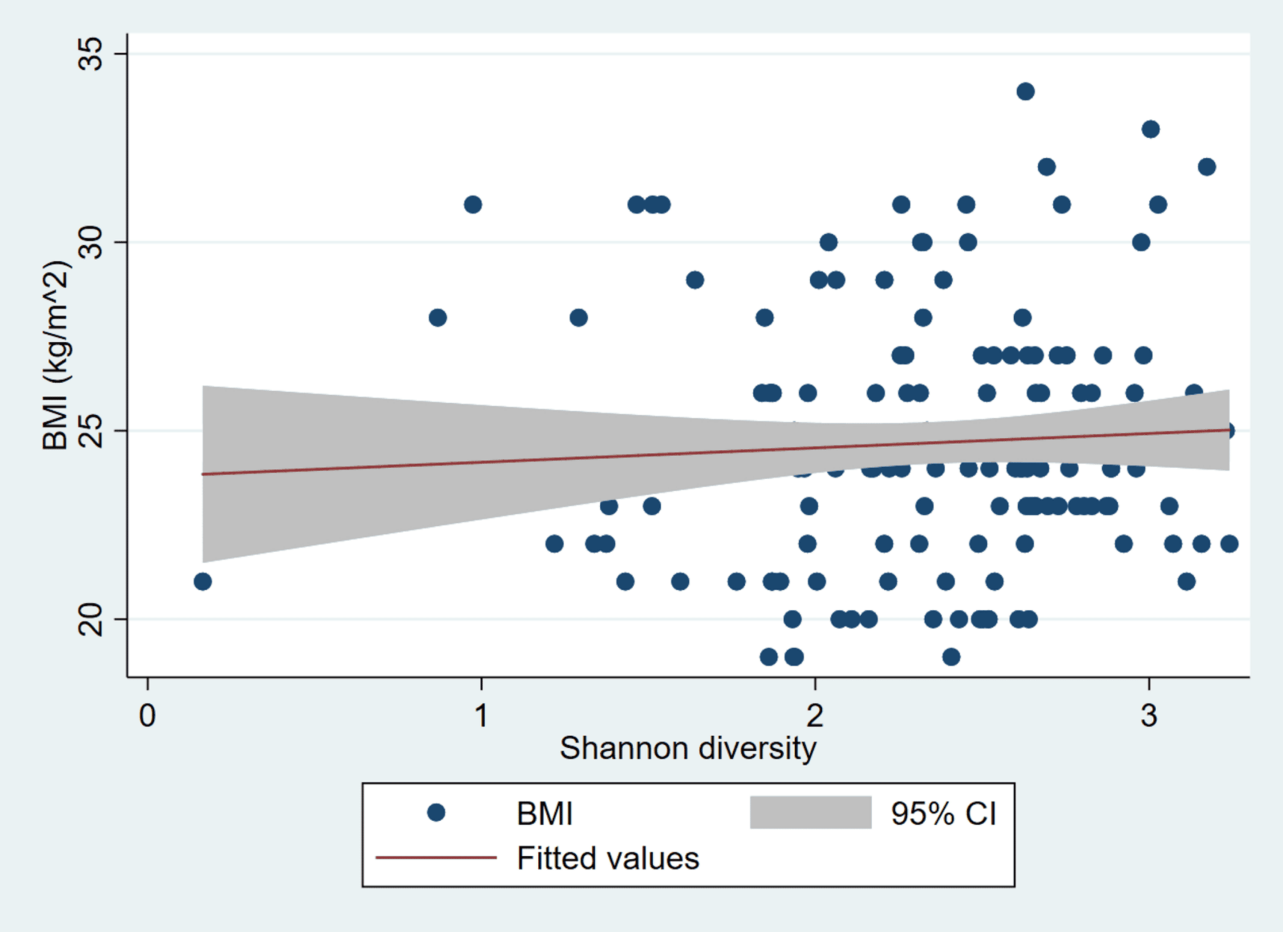
Scatterplot showing the relationship between body mass index and Shannon diversity index BMI: body mass index The figure illustrates the linear relationship between Shannon diversity and BMI among participants aged 18–39 years. Each point represents an individual participant, while the solid red line indicates the fitted regression line with the surrounding shaded area denoting the 95% confidence interval

In visual agreement with the regression analysis, the scatterplot demonstrates a weak, non-significant positive trend between Shannon diversity and BMI (Figure 1). Most data points are widely dispersed around the fitted regression line, suggesting considerable individual variability and no strong linear relationship between microbial diversity and body weight. The narrow slope of the fitted line and the broad 95% confidence band reinforce the statistical finding that Shannon diversity is not a meaningful predictor of BMI in this sample. Collectively, the graphical and statistical results indicate that within a relatively young, healthy adult population, gut microbial alpha-diversity does not exhibit a significant linear association with BMI.

## Discussion

This study investigated the association between gut microbiota alpha-diversity and BMI among healthy young adults (18-39 years) in the United States using CuratedMetagenomicData. The analysis revealed no significant relationship between microbial diversity and BMI, even after adjusting for demographic factors (age and gender). These findings suggest that, within a metabolically healthy young population, overall gut microbial diversity remains relatively stable and may not directly influence body mass. Instead, the functional activity of gut microbes and their interaction with host physiology may play a more meaningful role in metabolic regulation.

The gut microbiota communicates with brain centers governing hunger, stress, and metabolism through the microbiota-gut-brain axis (MGBA). This connection operates via neural, endocrine, and immune pathways, with the vagus nerve as a key mediator. Gut microbes produce SCFAs, such as acetate, propionate, and butyrate, which act on G-protein-coupled receptors (GPR41, GPR43) to stimulate gut hormones like glucagon-like peptide-1 (GLP-1), peptide YY (PYY), and glucose-dependent insulinotropic polypeptide (GIP). These hormones signal the hypothalamus to regulate appetite, satiety, and glucose balance. Microbial metabolites, including neurotransmitters such as gamma-aminobutyric acid (GABA) and serotonin (5-HT), further modulate the hypothalamic-pituitary-adrenal (HPA) axis, influencing mood and stress responses. Conversely, microbial components such as lipopolysaccharides can provoke inflammation, impair insulin signaling, and affect energy homeostasis. Collectively, these mechanisms underscore that microbial function, not merely diversity, drives the link between the gut, brain, and metabolism.

While diversity is generally associated with good health, it does not necessarily ensure microbial balance or functionality. Dysbiosis may occur even when diversity appears adequate if key metabolic functions are disrupted. Thus, evaluating microbial activity and metabolite production provides a more accurate reflection of metabolic resilience and gut health [6,11,12].

These results align with studies highlighting the value of investigating gut microbiota changes before disease onset. Somnuk et al. [17] reported that overweight but otherwise healthy young adults already exhibit indefinite metabolic and inflammatory disturbances compared to normal-weight peers. Davis et al. [18] observed that Western dietary patterns exert a stronger effect on gut microbial diversity than BMI itself, suggesting that diet may initiate early microbial shifts leading to metabolic risk. Similarly, Wang et al. [21] demonstrated that early evaluation of microbial and causal pathways helps clarify how gut variations influence BMI and metabolic health. Together, these findings emphasize that early detection of microbial and lifestyle-related changes can inform preventive strategies long before overt disease develops.

Lifestyle and medication exposures significantly influence the gut-microbiota ecosystem. Diets that are high in processed foods and low in fiber tend to limit microbial diversity and also impair functional capacity. In contrast, fiber-rich foods (whole grains, legumes, vegetables) combined with regular physical activity are associated with richer and more resilient microbiomes. For example, diet and exercise together have been shown to modulate gut microbiota diversity and metabolic signaling [16]. Likewise, in studies of healthy young adults, those with healthier lifestyle factors (fiber-rich diet with activity) exhibited higher microbial diversity and metabolic inflammatory profiles [17]. On the other hand, exposure to antibiotics, especially broad-spectrum or repeated treatments, can cause rapid loss of gut bacterial species, reduced richness, and persistently modified community composition that may impair metabolic, immune, and gut-brain signaling [18]. In summary, an unhealthy diet, low activity, and frequent antibiotic disruptions form a triad that can undermine microbial diversity and resilience. However, a fiber-rich diet, exercise, and avoidance of unnecessary antibiotics help preserve and safeguard it.

Strengths and limitations

The use of curated, standardized metagenomic data was a key strength, ensuring data consistency and analytical reliability. Focusing on a homogeneous, healthy young adult cohort minimized confounding from disease or medication effects. However, the study’s cross-sectional nature limits causal inference, and potential confounding by unmeasured lifestyle or dietary factors cannot be excluded. A major limitation was the absence of data related to the gut-brain-metabolic axis, including microbial metabolites and neuroendocrine indicators. This restricted exploration of the mechanistic pathways connecting gut diversity and metabolic regulation.

Recommendations

From a clinical and public-health standpoint, lifestyle modification remains central to maintaining healthy BMI and metabolic well-being. Physicians should emphasize balanced, fiber-rich, and minimally processed diets, coupled with regular physical activity, to support beneficial microbial activity and metabolic health. Since age influenced BMI in this study, age-specific dietary interventions, particularly those rich in plant-based nutrients and fermented foods, may help sustain gut-brain-metabolic balance.

At the community level, promoting exercise, reducing processed food intake, and increasing the consumption of vegetables and fermented foods can improve metabolic health outcomes. Parents are encouraged to foster healthy dietary habits early in childhood by providing balanced, nutrient-dense meals to support optimal microbiota function.

Finally, researchers should pursue longitudinal studies incorporating metagenomic, metabolomic, and hormonal data to deepen understanding of the MGBA. Such integrative approaches will clarify how microbial functions, not just diversity in shape body weight regulation, stress resilience, and overall metabolic health.

## Conclusions

This study investigated the association between gut microbiota alpha-diversity and BMI among healthy young adults in the United States using CuratedMetagenomicData. The findings showed no significant relationship between microbial diversity and BMI, even after adjusting for age and gender. These results suggest that, within a metabolically healthy young population, overall gut microbial diversity remains relatively stable and may not directly influence body mass. Instead, functional or compositional aspects of the microbiome could play a greater role in metabolic regulation. Future research incorporating longitudinal and multi-omic approaches is needed to better clarify the mechanisms linking the gut microbiota to the gut-brain-metabolic axis.

## References

[1] Akash MS, Fiayyaz F, Rehman K, Sabir S, Rasool MH (2019). Gut microbiota and metabolic disorders: advances in therapeutic interventions. Crit Rev Immunol.

[2] Nie X, Chen J, Ma X (2020). A metagenome-wide association study of gut microbiome and visceral fat accumulation. Comput Struct Biotechnol J.

[3] Xu Z, Jiang W, Huang W, Lin Y, Chan FK, Ng SC (2022). Gut microbiota in patients with obesity and metabolic disorders-a systematic review. Genes Nutr.

[4] Menni C, Zhu J, Le Roy CI (2020). Serum metabolites reflecting gut microbiome alpha diversity predict type 2 diabetes. Gut Microbes.

[5] Loftfield E, Herzig KH, Caporaso JG (2020). Association of body mass index with fecal microbial diversity and metabolites in the Northern Finland birth cohort. Cancer Epidemiol Biomarkers Prev.

[6] Bhalla D, Dinesh S, Sharma S, Sathisha GJ (2024). Gut-brain axis modulation of metabolic disorders: exploring the intertwined neurohumoral pathways and therapeutic prospects. Neurochem Res.

[7] Longo S, Rizza S, Federici M (2023). Microbiota-gut-brain axis: relationships among the vagus nerve, gut microbiota, obesity, and diabetes. Acta Diabetol.

[8] Han H, Yi B, Zhong R (2021). From gut microbiota to host appetite: gut microbiota-derived metabolites as key regulators. Microbiome.

[9] Romaní-Pérez M, Líebana-García R, Flor-Duro A, Bonillo-Jiménez D, Bullich-Vilarrubias C, Olivares M, Sanz Y (2025). Obesity and the gut microbiota: implications of neuroendocrine and immune signaling. FEBS J.

[10] Mitev K, Taleski V (2019). Association between the gut microbiota and obesity. Open Access Maced J Med Sci.

[11] Scheithauer TP, Rampanelli E, Nieuwdorp M, Vallance BA, Verchere CB, van Raalte DH, Herrema H (2020). Gut microbiota as a trigger for metabolic inflammation in obesity and type 2 diabetes. Front Immunol.

[12] Arifuzzaman M, Collins N, Guo CJ, Artis D (2024). Nutritional regulation of microbiota-derived metabolites: implications for immunity and inflammation. Immunity.

[13] Nizigiyimana P, Xu B, Liu L (2022). Gut microbiota is associated with differential metabolic characteristics: a study on a defined cohort of Africans and Chinese. Front Endocrinol (Lausanne).

[14] Andoh A, Nishida A, Takahashi K (2016). Comparison of the gut microbial community between obese and lean peoples using 16S gene sequencing in a Japanese population. J Clin Biochem Nutr.

[15] Koliada A, Syzenko G, Moseiko V (2017). Association between body mass index and Firmicutes/Bacteroidetes ratio in an adult Ukrainian population. BMC Microbiol.

[16] Campaniello D, Corbo MR, Sinigaglia M, Speranza B, Racioppo A, Altieri C, Bevilacqua A (2022). How diet and physical activity modulate gut microbiota: evidence, and perspectives. Nutrients.

[17] Somnuk S, Komindr S, Monkhai S, Poolsawat T, Nakphaichit M, Wanikorn B (2023). Metabolic and inflammatory profiles, gut microbiota and lifestyle factors in overweight and normal weight young thai adults. PLoS One.

[18] Davis SC, Yadav JS, Barrow SD, Robertson BK (2017). Gut microbiome diversity influenced more by the Westernized dietary regime than the body mass index as assessed using effect size statistic. Microbiologyopen.

[19] Pasolli E, Schiffer L, Manghi P (2017). Accessible, curated metagenomic data through ExperimentHub. Nat Methods.

[20] Schiffer L, Waldron L, Pasolli E (2023). Curated Metagenomic Data of the Human Microbiome. Bioconductor package version 3.21. https://bioconductor.org/packages/release/data/experiment/html/curatedMetagenomicData.html.

[21] Wang C, Ahn J, Tarpey T, Yi SS, Hayes RB, Li H (2023). A microbial causal mediation analytic tool for health disparity and applications in body mass index. Res Sq.

